# The Impact of Gestational Diabetes Mellitus on Human Milk Metabolic Hormones: A Systematic Review

**DOI:** 10.3390/nu14173620

**Published:** 2022-09-01

**Authors:** Majed A. Suwaydi, Xiaojie Zhou, Sharon L. Perrella, Mary E. Wlodek, Ching Tat Lai, Zoya Gridneva, Donna T. Geddes

**Affiliations:** 1 School of Molecular Sciences, The University of Western Australia, Crawley, WA 6009, Australia or; 2 Department of Medical Laboratory Technology, College of Applied Medical Sciences, Jazan University, Jazan 54142, Saudi Arabia; 3 Population Health, Murdoch Children’s Research Institute (MCRI), Parkville, VI 3052, Australia

**Keywords:** systematic review, gestational diabetes mellitus, human milk composition, metabolic hormones, infant, pregnancy, lactation, breastfeeding

## Abstract

Gestational diabetes mellitus (GDM) is a common pregnancy complication with short- and long-term health consequences for the infant and mother. Breastfeeding is the recommended mode of feeding as it offers an opportunity to reduce the risk of GDM consequences, likely partially mediated through changes in human milk (HM) composition. This review systematically reviewed 12 identified studies that investigated the impact of GDM on concentrations of HM metabolic hormones. Meta-analysis was not possible due to significant heterogeneity in study designs and hormone measurement techniques. The risk of bias was assessed using the National Institute for Clinical Excellence (NICE) tool. The methodological qualities were medium in half of the studies, while 25% (3/12) of studies carried a high risk of bias. Significant relationships were reported between GDM and concentrations of HM ghrelin (3/3 studies), insulin (2/4), and adiponectin (2/6), which may play an integral role in infant growth and development. In conclusion, preliminary evidence suggests that GDM may alter HM metabolic hormone concentrations; however, these relationships may be limited to the early lactation stage.

## 1. Introduction

Human milk (HM) is the optimal source of infant nutrition for achieving normal growth and development [1]. The WHO recommends exclusive breastfeeding in the first six months of infant life and continuation of breastfeeding for up to two years and beyond [2]. Being breastfed is linked to reduced risks of non-communicable diseases (NCDs) such as diabetes and cardiovascular disease later in life [3]. A series of maternal metabolic adaptations occur during pregnancy to cover fetal growth requirements, including glucose homeostasis adjustment. Slight increases in both insulin resistance and insulin synthesis are normal physiological pregnancy adaptations that enable the maintenance of maternal blood glucose levels whilst providing an adequate supply of glucose to the fetus through the placenta [4]. Dysregulation of this metabolic adaptation causes gestational diabetes mellitus (GDM) [5], which is associated with substantial short- and long-term risks for the infant [6]. As breastfeeding offers an opportunity to reduce maternal risks of developing obesity and diabetes [7,8], it is highly recommended that women with GDM breastfeed. Unfortunately, with increasing rates of maternal obesity [9], GDM prevalence [10] will likely continue to rise, as pre-pregnancy overweight/obesity and excessive gestational weight gain have been linked to GDM [11]. Currently, 16.2% of global live births are exposed to some degree of hyperglycemia, with 86.4% due to GDM [12].

The protective mechanism of breastfeeding for the infant is likely due to the unique bioactive composition of HM that is dynamic in response to maternal factors and the stage of lactation. An array of metabolic hormones such as adiponectin, leptin, ghrelin, insulin, apelin, and others have been recently identified in HM. These hormones act as signalling molecules and regulate metabolic activity in cells and tissues by binding to receptors which in turn transduce the signal to affect the metabolism of the cells and, evidently, have a role in programming the metabolism of the newborn infant as they are implicated in infant growth and development of body composition [1]. For example, both concentration and dose of HM adiponectin are implicated in infant feeding frequency and gastric emptying [13], and a higher 24-h intake of HM adiponectin is associated with lower infant fat-free mass and higher adiposity [14]. Thus, the links between HM metabolic hormones and the development of infant body composition emphasise early nutrition as a critical window of opportunity to influence metabolic programming with the potential to reduce the risk of later disease [15,16,17].

A limited number of studies have attempted to characterise the impact of GDM on HM hormones [18]. Recently Peila and colleagues reviewed the impact of both GDM and insulin-dependent type 2 diabetes mellitus (T2D) on an array of HM components, including macronutrients and bioactive molecules. The review identified 29 papers (21 on GDM and HM composition) and concluded that diabetes, including GDM, can alter HM composition and, specifically, HM metabolic hormones throughout lactation [18]. In addition to the wider scope, the review did not include a qualitative assessment (risk of bias) of the studies. Therefore, this study aims to systematically review the published evidence focusing on the relationships between GDM and HM metabolic hormones.

## 2. Materials and Methods

### 2.1. Protocol

The systematic review method was based on recommendations of the Preferred Reporting Items for Systematic Review and Meta-Analysis Protocols (PRISMA-P). The protocol has been registered on the International Prospective Register of Systematic Reviews database (PROSPERO), reference CRD42020192678. The study did not require ethical approval.

### 2.2. Search Strategy

The proposed literature search was initially performed in May 2020 and updated in July 2022 using the following electronic databases: MEDLINE, EMBASE, Cochrane Library, and Web of Science. Only human studies were included with no date range restrictions. The keyword terms, keywords, and medical subject headings (MeSH) used to conduct the search were (pregnant woman OR pregnant women OR female OR females OR woman OR women OR pregnancy) AND (pregnancy diabetes mellitus OR diabetes, gestational OR diabetes, pregnancy OR gestational diabetes OR gestational diabetes mellitus OR pregnancy diabetes OR pregnancy in diabetics) AND (breast milk OR breastfed infant OR human milk OR milk, human OR breast feeding OR breastfeeding OR feeding, breast OR breastfeeding OR colostrum OR lactation) AND (peptide hormone OR hormone OR hormones). The reviewers (MS and XJ) excluded any study that did not investigate HM metabolic hormones. The reference list of related studies was scanned, and a weekly search alert of the databases was set up to ensure up-to-date literature coverage. The last weekly search update was on 31 July 2022.

### 2.3. Eligibility Criteria

Our primary data showed a limited number of studies investigating the impact of GDM on HM metabolic hormones. Studies that met the following criteria were subsequently included: (1) data reported in the English language; (2) human studies with an epidemiological design: prospective, retrospective observational studies, including cross-sectional, comparative, and longitudinal studies. Conference abstracts, editorials, letters to the editor, case reports, and case series were excluded.

### 2.4. Selection Process

The search results were uploaded into Endnote 20 [19]. Duplications were removed, and then independently, two authors (M.A.S. and X.Z.) conducted the primary screening. The primary search outcomes were then uploaded into the JBI System for the Unified Management, Assessment and Review of Information (JBI SUMARI; JBI, Adelaide, Australia) to perform a secondary screening. The two authors performed a critical appraisal process independently using JBI SUMARI. Any disagreement between the two authors (M.A.S. and X.Z.) was resolved by mutual discussion or by involving a third author (Z.G.).

### 2.5. Data Extraction

The reviewers (M.A.S. and X.Z.) obtained, read the full texts of all potentially relevant articles, and independently extracted data from each selected article. A standard data extraction form was used to minimise inconsistency between reviewers. Data included author and year of publication, descriptive information about the study design, country and setting, baseline characteristics of the study population, methodology of milk sampling, timing of sample collection, method of assessment of hormones concentration, outcome definition, time of outcome assessment and details of statistical analysis. A third reviewer (Z.G.) decided on the outcome in case of disagreement.

### 2.6. Quality Assessment

The risk of bias was assessed independently by two authors (M.A.S. and X.Z.) using the National Institute for Clinical Excellence (NICE) methodological checklist for cohort studies [20], and a final score was obtained by agreement after discussion between the three authors (M.A.S., X.Z., and Z.G.).

## 3. Results

### 3.1. Synthesis

**Figure 1:**
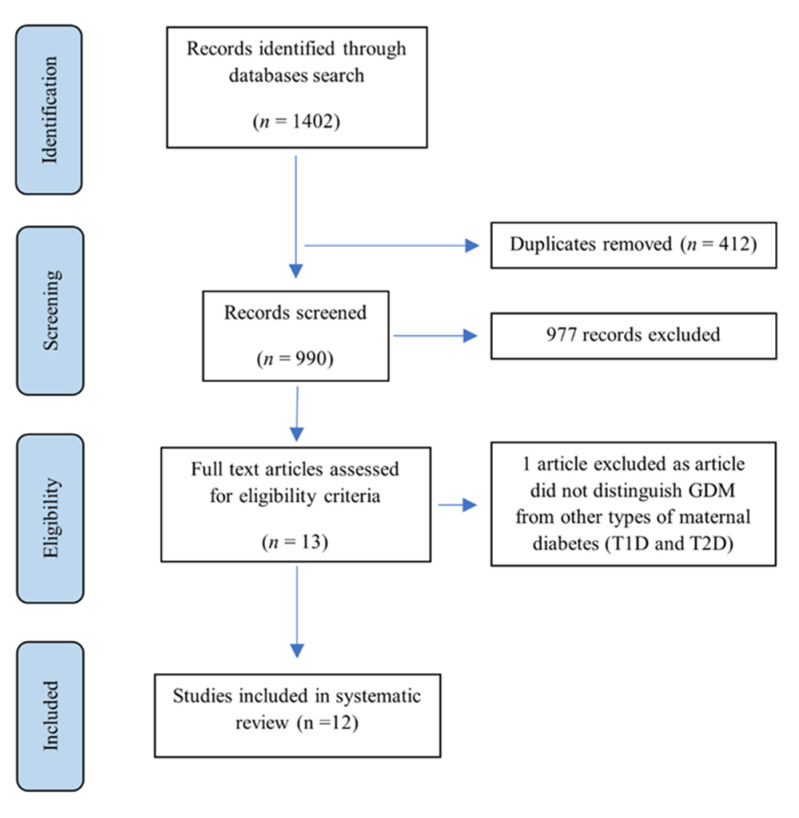
PRISMA diagram of the studies of the systematic search and studies included. GDM, gestational diabetes mellitus; T1D, type 1 diabetes; T2D, type 2 diabetes.

A total of 1402 articles were identified based on the search strategy. After removing duplicates (*n* = 412), 990 titles/abstracts were screened for eligibility. Of these, 13 articles were eligible for a full-text assessment. One study was excluded as the sample included mothers with different types of diabetes (Type 1 diabetes (T1D), T2D, and GDM), and the HM analytical method was not reported. In total, 12 studies were included in the systematic review. The PRISMA diagram of the systematic search and included studies are presented in Figure 1.

### 3.2. Description of Studies

The 12 studies included in this systematic review were published between 2007 and 2022 (Table 1). All included papers were classified as prospective observational longitudinal studies [21,22,23,24,25,26,27,28,29,30,31,32].

### 3.3. Participant Characteristics

Studies included participants from eight countries, with most conducted in Turkey [21,22,24,25,29], and the remainder conducted in Brazil [26], Canada [23], China [27], Finland [30], New Zealand [31], Pakistan [28], and the United States of America [32]. Cohort sizes ranged from 20 to 510; however, the number of participants in the GDM group ranged from 10 to 48 (Table 1).

### 3.4. Stage of Lactation

**Figure 2:**
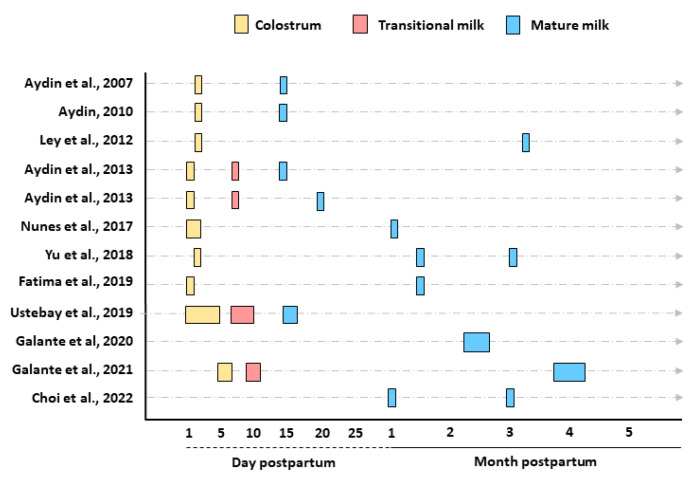
Timing of human milk sample collection.

The times and categories of HM sample collection were reported as 1–5 days (colostrum), 6–14 days (transitional milk), and ≥15 days (mature milk) [1] (Figure 2). Colostrum and mature milk were collected in six studies [21,22,23,26,27,28]. Colostrum, transitional, and mature milk were collected in four studies [24,25,29,31], and only mature milk was collected in two studies [30,32].

### 3.5. Human Milk Sample Collection, Storage, and Preparation for Analysis

Limited data were reported regarding sample collection with respect to the time of the day or pre- and post-breastfeed/expression (Table 1). Two studies did not report the time of sample collection [23,26]. Morning sample collection was reported for the remaining 10 studies [21,22,24,25,27,28,29,30,31,32]; 5 of which did not specify the time of collection but reported morning collection after an overnight fast [21,22,24,25,29], and 7 studies did not report maternal fasting status [23,26,27,28,30,31,32]. In addition, one study reported different times of sample collection between colostrum and mature milk (colostrum samples collected in the morning and mature milk samples collected in the afternoon) [27].

Skimmed milk samples were prepared for analysis in all 12 studies. Sample storage temperatures were reported in 11 studies as −40 °C [24], −70 °C [21,30], and −80 °C [23,25,26,27,28,29,31,32] (Table 1). However, limited data were provided on how samples were maintained during transportation and preparation; one study reported collecting samples using tubes containing aprotinin or Tween-20 [25], and one study reported adding aprotinin before analysis [21].

### 3.6. Measurement of Human Milk Metabolic Hormones

Enzyme-linked immunosorbent assay (ELISA) was used in the majority of studies (11/12) [22,24,25,26,27,28,29,30,31,32]. Other techniques included radioimmunoassay (RIA) [21,23], enzyme immunoassay (EIA) [22,24], high-pressure liquid chromatography (HPLC) [21], and electrochemiluminescence [23].

### 3.7. Statistical Analysis

Studies included in this systematic review can be classified into two categories based on their statistical approach: (a) statistical tests used to determine if there were differences between the comparative groups (GDM vs. non-GDM); (b) statistical tests used to determine the relationship (correlation/association) between GDM and HM composition (Table 2). Mann–Whitney U test [21,24,25,28,29], *t*-test [29], Kruskal–Wallis test [26], and multivariate analysis of variance (MANOVA) [30] were used to identify differences between GDM and non-GDM groups. The Spearman’s rank-order correlation [22], general linear models [23], generalised estimating equation (GEE) [27], and mixed-effect modelling [31,32] were used to test for associations/correlations between GDM status and HM components. While the total sample size was considered large in the majority of the 12 studies, sample sizes of GDM sub-groups were small, with a mean sample size of 26.7 ± 13.9 (Table 1), and sample size power calculation was reported for only 1 study [30]. Limited information was provided on the proportions of missing data among the variables or the methods used to handle missing data. Only five studies adjusted for potential confounders, including maternal age, body mass index (BMI), ethnicity, breastfeeding status, and time elapsed from birth to milk collection [23,27,28,30,32], and three studies adjusted for multiple comparisons [27,30,31].

### 3.8. Gestational Diabetes Mellitus and Human Milk Metabolic Hormones

**Figure 3:**
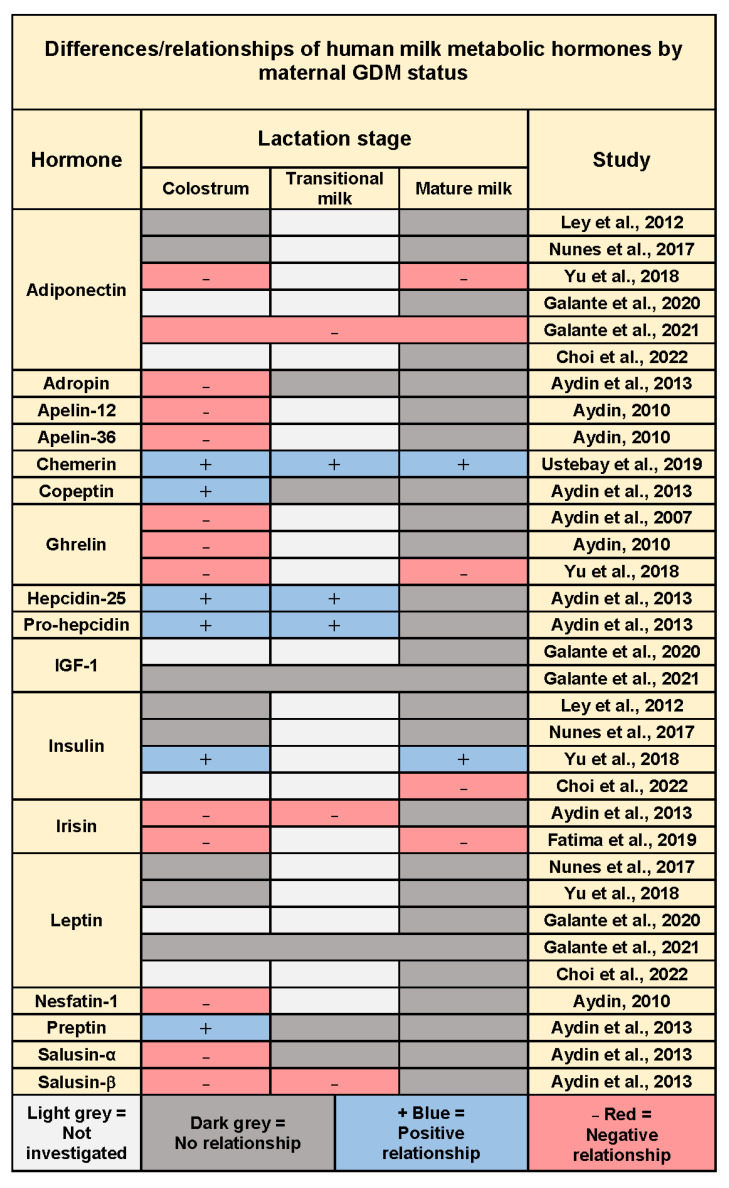
Summary of results of quantitative synthesis across stages of lactation for studies investigating differences between GDM and control group and/or relationships between GDM and concentrations of human milk hormones at different lactation stages (*p* < 0.05). Galante et al., 2021 combined concentration results at all time points for analysis with no time effect reported.

Twelve studies explored relationships between maternal GDM and concentrations of HM metabolic hormones. These hormones include but are not limited to adiponectin, leptin, insulin, ghrelin, and irisin (Table 1 and Figure 3).

Six studies investigated HM adiponectin in relation to maternal GDM status. Two studies found negative relationships at various stages of lactation in milk after term [27] and preterm birth [31]. Four studies reported no relationship [23,26,30,32]. Interestingly, one of them [30], whilst finding no overall difference between GDM and the control group, indicated that infant sex might contribute to the relationship between maternal GDM status and HM adiponectin, as milk of women with GDM that gave birth to male infants had the lowest concentration of adiponectin compared to both women with GDM that gave birth to female infants and women without GDM independent of infant sex. However, the sex-specific differences became non-significant when correcting for the duration of exclusive breastfeeding (*p* = 0.05), and the control group in this study was more than tenfold larger than the GDM group [30].

Five studies investigated HM leptin, one at all three stages of lactation [31], two in colostrum and mature milk [26,27] and two in mature milk [30,32]. All found no difference in HM leptin concentration in relation to maternal GDM status.

Four studies reported findings on HM insulin; two found no difference by GDM status in colostrum and mature milk [23,26], and one found increased HM insulin in both colostrum and mature milk [27]. However, the fourth study found a reduction in mature HM insulin in GDM group mothers [32].

Three studies focused on HM ghrelin and found lower concentrations in colostrum [21,22,27] but not in mature milk [21,22].

HM irisin was investigated in two studies, and decreased concentrations were found in colostrum [24,28], transitional [24], and mature milk [28].

Two studies that investigated IGF-1 concentration reported no differences according to maternal GDM status [30,31].

In addition, four studies were the first to measure such hormones in HM as apelin, nesfatin-1, copeptin, adropin, preptin, salusin α and β, pro-hepcidin, hepcidin-25, and chemerin and investigate differences in regard to GDM status [22,24,25,29]. Increases in concentrations were reported for the early lactation stage for copeptin, preptin, pro-hepcidin, hepcidin-25, and chemerin, and decreased concentrations for nesfatin-1, apelin-36, adropin, and salusin α and β. These differences were not apparent in mature milk except for chemerin, which was increased with GDM (Table 1, Figure 3).

### 3.9. Risk of Bias

**Figure 4:**
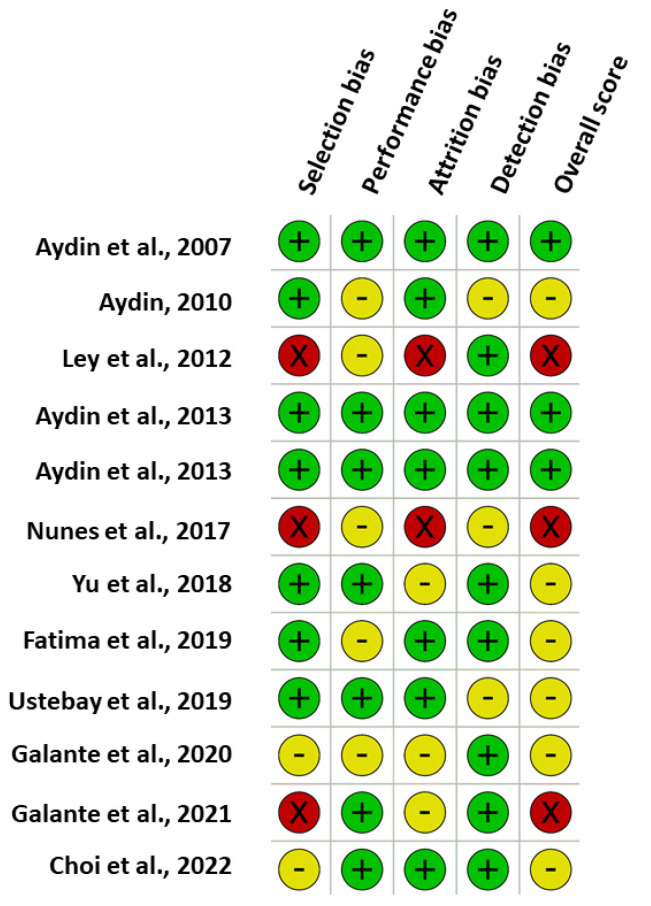
Risk of bias in studies assessing the relationship between concentrations of metabolic hormones in human milk and gestational diabetes mellitus using the National Institute for Clinical Excellence methodological checklist. “+, green,” low risk of bias; “×, red,” high risk of bias; “-, yellow,” unclear/medium risk of bias.

Evaluation of the quality of the studies included in this systematic review found that the majority had various types of bias, predominantly attrition and detection bias. The risk of bias was assessed to be high in three studies (25%), medium in six studies (50%) and low in three studies (25%) (Table A1, Figure 4).

## 4. Discussion

The rising incidence of GDM is of increasing interest with regard to its effect on maternal and infant health as well as its influence on HM composition. This systematic review summarises the findings of 12 studies that investigated the impact of GDM on HM metabolic hormones. We employed a robust strategy to search and synthesise evidence, provide critical analysis, and rate the quality of current literature, highlighting both the paucity of research and the need for more in-depth investigation in the future. We provide a comprehensive overview of the reported differences in HM metabolic hormones with GDM. To date, data from the included studies reveal a lack of evidence to develop a clear understanding of how GDM might influence the hormonal composition of HM. While there is limited evidence, the quantitative synthesis indicates lower concentrations of HM adiponectin, ghrelin, and irisin among women with GDM, particularly during the establishment of lactation. Additionally, limited evidence points to altered concentrations for some other metabolic hormones in colostrum, transitional, and mature HM of women with GDM. However, the considerable heterogeneity in study design and methodology makes it difficult to draw robust conclusions.

The major findings of this systematic review point to limitations in the literature that underline the need to further investigate the impact of GDM on HM metabolic hormones. Only 12 eligible studies were identified; most of the studies were of exploratory nature with small sample sizes, heterogeneous study designs and reporting of outcomes (including concentrations) that excluded the possibility of meta-analysis. However, the quantitative synthesis (Figure 3) suggests GDM might indeed be an indicator of concentrations of HM metabolic hormones.

Adiponectin, the most abundant hormone in HM, was analysed in six studies, and two reported lower concentrations of HM adiponectin after a GDM-complicated pregnancy [27,31]. A previous report indicated that a higher intake of HM adiponectin was associated with both lower infant lean body mass and higher adiposity [14]. Circulating adiponectin has previously been reported to be substantially lower in women with GDM compared with those without GDM [33], and this might, in part, contribute to lower HM adiponectin observed with GDM. As low serum adiponectin is also associated with central obesity and metabolic syndrome [34], future studies should account for both maternal BMI and infant 24 h milk intake to determine possible impacts on infant growth and development.

To date, four studies have assessed the effect of GDM on HM insulin [23,26,27,32]. Only one study found a significant increase in HM insulin; however, the participants initially received dietary treatment, and those who did not respond to dietary intervention (*n* = 17) received insulin therapy. The difference in HM insulin between the GDM diet treatment group and the healthy group was not significant in both colostrum and mature milk [27]. Nunes et al. [26] indicated no significant difference between GDM and the control group in a study that included only mothers with diet-controlled GDM. It is important to note that, in Nunes et al. [26], maternal pre-pregnancy BMI was not significantly different between the GDM and control groups, and the sample size was considerably small. Despite no significant differences being documented, pre-pregnancy BMI > 25 kg/m^2^, insulin resistance, and insulin sensitivity were associated with higher HM insulin [23]. Herein, maternal BMI and pregnancy hyperglycaemia seem to be important factors modulating the levels of HM insulin in GDM mothers. These issues are highlighted in a recent report by Choi et al. [32], where HM insulin concentration was lower in women with GDM compared with a control group using multiple adjusted models, including pre-pregnancy BMI, gestational weight gain, and postpartum weight loss. The conflicting findings between studies may, however, also be a result of postpartum heterogeneities in cohorts’ characteristics, as Yu et al. [27] excluded women with postpartum glucose abnormalities. Therefore, future work must consider pre-pregnancy and pregnancy parameters, including maternal BMI/adiposity, hyperglycaemia, insulin resistance, and insulin sensitivity, as confounding factors in the relationship between GDM and HM insulin.

In three studies, HM ghrelin concentration was lower in the colostrum of women with GDM [21,22,27], and, despite the compositional differences between colostrum and mature milk, a study that collected mature milk reported a similar result [27]. Maternal serum ghrelin concentration is reported to be lower after GDM-complicated pregnancy; therefore, the lower HM ghrelin concentration is likely a reflection of the corresponding maternal serum ghrelin concentration [21,35].

Irisin was first identified in HM in 2013 [24], and the mechanisms by which irisin presents in milk have not yet been elucidated. Two studies have reported significantly lower concentrations of HM irisin after GDM-complicated pregnancy [24,28], supported by a recent systematic review that reported significantly lower concentration of circulating irisin in pregnant women with GDM, which normalised to that of women without GDM after giving birth [36]. Aydin et al. [24] reported a similar trend in that both HM and maternal circulating irisin concentrations increased from colostrum to mature milk, and significant differences in HM irisin concentrations between women with and without GDM were only detected in colostrum and transitional but not mature milk. This result, in part, conflicted with Fatima et al. [28], which reported significantly lower maternal circulating irisin concentrations pre- and postpartum and also in colostrum and mature milk of women with GDM compared to women without GDM, irrespective of lactation stage. Authors have speculated that continued breastfeeding with lower concentrations of HM irisin may negatively affect infant health and lipid regulation resulting in higher adiposity [28]. However, that conclusion cannot be made without measuring infant 24 h milk intake and intake of irisin. Additionally, unlike Aydin et al. [24], who did not find significant BMI differences between GDM and non-GDM groups, Fatima et al. [28] accounted for maternal BMI (significantly different between the groups), which nullified significant correlations between HM irisin, maternal GDM status, and infant weight at birth and 6 weeks postpartum. Thus, the impact of GDM on HM irisin and its potential influence on infant growth and body composition requires further longitudinal investigation, accounting for maternal adiposity and HM infant intake.

Maternal serum leptin concentration is reported to be higher in women with GDM [37], irrespective of maternal BMI [38]. Indeed, maternal plasma leptin enters HM via diffusion or receptor-mediated transport [39,40,41] or lactocyte secretion [42]; therefore, increasing maternal plasma leptin due to adiposity [43] or GDM might result in higher HM leptin. Despite that, none of the four studies that investigated HM leptin concentration found any differences with GDM [26,27,30,31,32]; this may be impacted by the measurement of HM leptin in skim milk, which has been shown to be considerably lower than that of whole milk [44]. Future studies of HM leptin in GDM women should therefore include an analysis of whole HM as a previous report indicated higher intake of whole HM leptin was associated positively with infant adiposity over the first 12 months of lactation [14].

HM IGF-1 concentration was reported not to differ according to GDM status in two studies [30,31]. This is unexpected due to the assumption that maternal circulating IGF-1 seems to be the primary source of HM IGF-1, and higher concentrations of serum IGF-1 have been reported in pregnant diabetic women compared with those without diabetes [45,46]. However, these findings require further investigation, assuming there may be other factors that might influence HM IGF-1 concentration.

Four studies [22,24,25,29] have measured lower concentrations of HM hormones adropin, apelin-12 and apelin-36, nesfatin-1, and salusin α and β, and higher concentrations of chemerin, copeptin, hepcidin-25, pro-hepcidin, and preptin, in the colostrum of women that had GDM [22,24,25]; chemerin was the only hormone reported to be higher in mature milk of GDM mothers [29]. However, further investigation with a larger sample size and controlling for maternal BMI is needed.

In summary, the impact of GDM on HM hormone concentrations still remains inconclusive, with differences found predominantly in colostrum and transitional milk in studies with low participant numbers and short follow-up (<one month postpartum). The results from HM milk composition studies, however, indicate that it is unlikely for HM components to change in the same longitudinal manner [40]. However, whether the longitudinal changes in metabolic hormone concentrations among mothers with GDM may contribute to losing the association at the mature lactation stage requires further longitudinal investigation.

Further, mothers with GDM may experience lactation difficulties such as delayed secretory activation and reduced milk production [47], which may lead to study withdrawal, contributing to biased results based on mothers with normal lactation outcomes; thus, the effect might not be seen. With reports of both higher and lower concentrations of several metabolic hormones discussed here, as well as of macronutrients [18] and the abundances of exosomal microRNAs [48], it is speculated by some authors that GDM-altered HM composition raises concerns about the negative effect on infant outcomes. Whilst exposure to GDM in utero increases the infant’s risk of T2D and obesity later in life [49], being breastfed to at least 3 months of age results in decreased risk [50,51]. Based on that, the impact of GDM on infant outcomes, such as body composition, via HM cannot be speculated upon without measuring HM intake and intake of HM components by the infant, particularly when considering only a few components. Further consideration should also be given to the differences in infant body composition existing at birth [52].

The interpretation of published data from these studies is problematic due to heterogeneity in study design, sample collection, preparation, and analysis which together may influence the outcomes of HM hormones studies [53]. Few studies collected sufficient information to provide a detailed insight into the impact of GDM on HM hormone concentration [27,28]. Sample sizes were low in most studies, and those with relatively large sample sizes had strongly unbalanced sample sizes between groups [23,26,30,31], potentially resulting in insufficiently powered studies to detect a difference between GDM vs. non-GDM groups. The impact of GDM on HM hormones may be closely related to maternal glycaemic status; therefore, future investigations should simultaneously measure and account for confounding factors such as maternal serum glucose concentration and adiposity. Only four studies have accounted for possible confounding factors [23,27,28,30], and no study accounted for maternal glycaemic status at the time of HM concentration assessment. Selection and attrition bias are potential issues for several of the observational studies reviewed (Figure 4).

This work is the first comprehensive systematic review of studies on the impact of GDM on HM metabolic hormones. A recent review by Peila et al. [18] that had an expansive focus on both GDM and T2D and an array of HM components concluded that both GDM and T2D could alter HM composition, including HM hormones. However, the review pointed out that considerable study limitations, specifically, evaluation of only a few specific HM components, may limit the ability to identify differences in HM composition, particularly between the acute nature of GDM compared to T2D on HM composition. The main strength of our analysis is that we conducted a broad search strategy to capture the largest possible number of publications, along with a rigorous approach to quality assessment that was performed in accordance with current guidelines. Peila et al. identified 9 papers on GDM that reported on 16 HM hormones and hormone-like bioactive molecules, whilst we have reported results for 17 HM components (including leptin) from 12 papers. In addition, systematic data extraction of the studies’ design and methodologies were performed to identify overarching concepts that are largely dismissed in previous literature, i.e., postpartum glycemic status, stage of lactation, time of sample collection, and pre-analytical and analytical methods consideration. However, this systematic review has some data-related limitations. The number of studies conducted is small, and a meta-analysis could not be conducted due to methodological heterogeneity among the studies.

## 5. Conclusions

Our systematic review of the current literature found there are limited studies that have investigated the impact of GDM on HM metabolic hormones composition. At present, these studies serve as preliminary evidence of the possible impact of GDM on HM metabolic hormones. High-quality, larger sample size studies with standardised methods of sample collection and validated methods of HM composition analysis are needed to obtain a comprehensive understating of the true effect of this pathology on HM hormones.

## Figures and Tables

**Table 1:** Summary of studies examining human milk metabolic hormones from lactating women who have had gestational diabetes mellitus.

Study	Country, Year, Cohort Size (*n*)	Sample Size/Group	Birth Gestation, Postpartum Glycemic Status ^a^	Lactation Stage (Timing of Sample Collection)	Collection Time, Method, (Storage Temperature)	Hormones Measured	Analytical Method	GDM Outcome Reported	Concentration, Mean ± SD, Median [IQR], Mean Difference (95% CI), and/or β (SEE), *p*-Value
Aydin et al., [21]	Turkey, 2007 (*n* = 34)	GDM = 12 T2D = 3 CTL = 14	Term, no	C (2 d) MM (15 d)	Fasting am Pre-feed, NR (−70 °C)	Ghrelin	RIA, HPLC	GDM: two-fold ↓ C acylated (active) ghrelin No difference in MM	Acyl-ghrelin C (fmol/mL), GDM: 7.75 ± 2.2; non-GDM: 18.99 ± 2.7 (*p* < 0.05) Acyl-ghrelin MM (fmol/mL), GDM: 16.06 ± 3.2; non-GDM: 16.47 ± 3.3
Aydin, [22]	Turkey 2010 (*n* = 20)	GDM = 10 CTL = 10	Term, no	C (2 d) MM (15 d)	Fasting am NR (NR)	Ghrelin Apelin-12 Apelin-36 Nesfatin-1	EIA: apelin-12, apelin-36 ELISA: ghrelin, nesfatin-1	GDM: ↓ C ghrelin, apelin-12, apelin-36, nesfatin-1 No difference in MM	Acyl-ghrelin C (pg/mL), GDM: 27.7 ± 2; non-GDM: 39.2 ± 2.0 (*p* < 0.05) Acyl-ghrelin MM (pg/mL), GDM: 37.7 ± 3.0; non-GDM: 48.2 ± 5.1 Des-acyl ghrelin C (pg/mL), GDM: 338.1 ± 49; non-GDM: 466.1 ± 52 (*p* < 0.05) Des-acyl ghrelin MM (pg/mL), GDM: 359.1 ± 51.2; non-GDM: 505.1 ± 52 (*p* < 0.05) Apelin-12 C (ng/mL), GDM: 2.9 ± 0.6; non-GDM: 4.3 ± 1.2 (*p* < 0.05) Apelin-12 MM (ng/mL), GDM: 3.6 ± 1.2; non-GDM: 5.4 ± 1.8 Apelin-36 C (ng/mL), GDM: 3.2 ± 0.7; non-GDM: 4.9 ± 2.0 (*p* < 0.05) Apelin-36 MM (ng/mL), GDM: 4.4 ± 1.4; non-GDM: 6.2 ± 1.9 Nesfatin-1 C (ng/mL), GDM: 0.78 ± 0.3; non-GDM: 1.6 ± 0.2 (*p* < 0.05); Nesfatin-1 MM (ng/mL), GDM: 0.98 ± 0.3; non-GDM: 1.2 ± 0.4
Ley et al., [23]	Canada, 2012 (*n* = 170)	GDM = 37 CTL = 133	Term, no	C (median 2 d (1, 3)) MM (median 95 d (91, 102))	NR C: HE or EBP, MM: complete breast expression from both breasts with EBP (−80 °C)	Adiponectin Insulin	RIA: adiponectin ECLIA: insulin	C, MM adiponectin and insulin not associated with GDM In the analysis restricted to C associations remained non-significant	Concentrations within groups NR Adiponectin C: −0.129 (0.180) (*p* = 0.47) Adiponectin MM: −0.081 (0.117) (*p* = 0.49) Insulin C: −0.200 (0.256) (*p* = 0.44) Insulin MM: 0.102 (0.174) (*p* = 0.56)
Aydin et al., [24]	Turkey, 2013 (*n* = 44)	GDM = 15 CTL = 15	Term, no	C (1 d) TM (7 d) MM (15 d)	Fasting am Pre-feed, NR (−40 °C)	Copeptin Irisin Adropin	EIA: copeptin ELISA: irisin, copeptin	GDM: ↑ C copeptin and adropin, ↓ C, TM irisin No difference in MM	For all hormones, results are reported as figures only (*p* < 0.05)
Aydin et al., [25]	Turkey, 2013 (*n* = 36)	GDM = 12 CTL = 12	Term, no	C (1 d) TM (7 d) MM (20 d)	Fasting am Pre-feed, NR (−80 °C)	Preptin Salusin-α Salusin-β Pro-hepcidin Hepcidin-25	ELISA	GDM: ↑ C preptin, ↓ C salusin-α and salusin-β, ↑ C, TM pro-hepcidin and hepcidin No difference in MM	Preptin C (ng/mL), GDM: 14.32 ± 3.06; non-GDM: 9.72 ± 2.26 (*p* < 0.05) Preptin TM (ng/mL), GDM: 11.72 ± 2.34; non-GDM: 9.02 ± 0.88 Preptin MM (ng/mL), GDM: 10.16 ± 2.19; non-GDM: 11.16 ± 5.70 Salusin-α C (pg/mL), GDM: 187.80 ± 19.01; non-GDM: 261.40 ± 31.35 (*p* < 0.01) Salusin-α TM (pg/mL), GDM: 211.20 ± 44.61; non-GDM: 242.20 ± 23.97 Salusin-α MM (pg/mL), GDM: 248.80 ± 22.14; non-GDM: 218.60 ± 60.0 Salusin-β C (pg/mL), GDM: 379.0 ± 100.86; non-GDM: 530.20 ± 70.18 (*p* < 0.05) Salusin-β TM (pg/mL), GDM: 425.0 ± 34.07; non-GDM: 494.40 ± 45.99 (*p* < 0.05) Salusin-β MM (pg/mL), GDM: 501.0 ± 65.60; non-GDM: 450.0 ± 68.04 Pro-hepcidin C (pg/mL), GDM: 814.0 ± 72.98; non-GDM: 649.60 ± 39.34 (*p* < 0.01) Pro-hepcidin TM (pg/mL), GDM: 761.40 ± 40.45; non-GDM: 572.0 ± 49.70 (*p* < 0.01) Pro-hepcidin MM (pg/mL), GDM: 613.60 ± 61.77; non-GDM: 528.80 ± 47.77 Hepcidin-25 C (pg/mL), GDM: 835.80 ± 93.73; non-GDM: 595.0 ± 77.26 (*p* < 0.01) Hepcidin-25 TM (pg/mL), GDM: 746.20 ± 82.18; non-GDM: 580.60 ± 82.76 (*p* < 0.05) Hepcidin-25 MM (pg/mL), GDM: 641.20 ± 63.71; non-GDM: 614.0 ± 63.85
Nunes et al., [26]	Brazil, 2017 (*n* = 69)	GDM = 12 CTL = 21	Term, no	C (1–2 d) MM (30 d)	NR HE, no control for pre-/post-feed sampling and maternal fasting status (−80 °C)	Adiponectin Insulin Leptin	ELISA	No difference between women with and without GDM	Adiponectin C (ng/mL), GDM: 10.23 [5.63, 22.65]; non-GDM: 8.79 [6.90, 11.35] Adiponectin MM (ng/mL), GDM: 12.43 [6.90, 14.87]; non-GDM: 9.87 [6.33, 11.50] Insulin C µIU/mL, GDM: 49.37 [25.70, 176.54]; non-GDM: 55.04 [11.57, 162.64] Insulin MM, GDM: 22.83 [16.33, 60.43]; non-GDM: 22.03 [13.30, 32.21] Leptin C (ng/mL), GDM: 0.67 [0.45,1.31]; non-GDM: 0.81 [0.42, 1.27] Leptin MM, GDM: 0.46 [0.45, 0.70]; non-GDM: 0.72 [0.49, 0.90]
Yu et al., [27]	China, 2018 (*n* = 96)	GDM = 48 CTL = 48	Term, yes	C (3 d) MM (42, 90 d)	3d: 8:00–9:00 Pre-feed 42d, 90d: 14:00–16:00 One breast expression with EBP (−80 °C)	Adiponectin Insulin Leptin Total ghrelin	ELISA	GDM: ↓ adiponectin and total ghrelin, ↑ insulin in C and at d90 No difference in hormone concentrations between women with and without GDM at d42 No difference in leptin concentrations between women with and without GDM	Adiponectin C (log ng/mL), GDM: 21.74 [14.77, 56.10]; non-GDM: 65.81 [29.76, 126.91] (*p* < 0·001) Adiponectin d42 (log ng/mL), GDM: 11.89 [8.0, 18.37]; non-GDM: 12.22 [9.69, 14.92] (*p* = 0.89) Adiponectin d90 (log ng/mL), GDM: 11.75 [8.53, 13.91]; non-GDM: 15.31 [11.60, 19.53] (*p* = 0.009) Insulin C (log µIU/mL), GDM: 22.80 [13.51, 51.25]; non-GDM: 20.41 [7.68, 31.38] (*p* = 0.047) Insulin d42 (log µIU/mL), GDM: 32.36 [13.06, 58.22]; non-GDM: 28.20 [17.97, 40.05] (*p* = 0.38) Insulin d90 (log µIU/mL), GDM: 40.63 [22.48, 57.17]; non-GDM: 24.61 [13.40, 31.85] (*p* = 0.021) Leptin C (log µIU/mL), GDM: 1.28 [0.87, 2.63]; non-GDM: 1.49 [0.56, 3.25] (*p* = 0.77) Leptin d42 (log µIU/mL), GDM: 0.26 [0.09, 0.47]; non-GDM: 0.21 [0.09, 0.51] (*p* = 0.69) Leptin d90 (log µIU/mL), GDM: 0.20 [0.12, 0.47]; non-GDM: 0.25 [0.16, 0.45] (*p* = 0.54) Total ghrelin C (log pg/mL), GDM: 124.43 [89.87, 178.76]; non-GDM: 159.36 [122.62, 234.33] (*p* = 0·011) Total ghrelin d42 (log pg/mL), GDM: 338.74 [189.98, 432.95]; non-GDM: 337.60 [149.82, 565.77] (*p* = 0.80) Total ghrelin d90 (log pg/mL), GDM: 104.62 [72.72, 154.71]; non-GDM: 210.91 [147.25, 381.88] (*p* < 0.001)
Fatima et al., [28]	Pakistan, 2019 (*n* = 66)	GDM = 33 CTL = 33	NR, no	C (1–3 d) MM (42 d)	08:00–10:00 2 h after previous breastfeed with manual breast pump (−80 °C)	Irisin	ELISA	GDM: ↓ irisin in C and MM	Irisin C (pg/mL), GDM: 10.36 ± 4.73; non-GDM: 57.08 ± 8.28 (*p <* 0.001) Irisin MM (pg/mL), GDM: 15.35 ± 0.42; non-GDM: 56.40 ± 9.55 (*p* < 0.001)
Ustebay et al., [29]	Turkey, 2019 (*n* = 60)	GDM = 26 CTL = 27	Term, no	C (1–5 d) TM (7–10 d) MM (15–17 d)	Fasting am NR (−80 °C)	Chemerin	ELISA	GDM: ↑ chemerin in C and MM	Results are reported as figure only (*p* < 0.05)
Galante et al., [30]	Finland, 2020 (*n* = 510)	GDM = 44 CTL = 460	Term 95.2%; Preterm 4.2%, no	MM (2.6 ± 0.4 mo)	10:00–12:00 HE full single breast, first few drops of milk discarded (−70 °C)	Adiponectin IGF-1 Leptin	ELISA	No overall difference between women with and without GDM ↓ MM adiponectin in GDM with male infant compared to GDM with female infant or CTL	Adiponectin MM (log 10 ng/mg): −0.012 [−0.099, 0.074] (*p* = 0.78) IGF-1 MM (log 10 ng/mg): 0.021 [−0.048, 0.091] (*p* = 0.55) Leptin MM (log 10 ng/mg): −0.018 [−0.093, 0.058] (*p* = 0.65) Sex-specific differences (*p* = 0.031) non-significant when correcting for exclusive duration of breastfeeding (*p* = 0.05)
Galante et al., [31]	New Zealand, 2021 (*n* = 194)	GDM = 36 CTL = 155	Preterm, no	C (5 ± 2 d) TM (10 ± 2 d) MM (4 ± 0.5 mo)	10:00–12:00 2–3 h after previous expression or breastfeed, complete right breast expression with EBP (−80 °C)	Adiponectin IGF-1 Leptin	ELISA	GDM: ↓ adiponectin independent of collection time point	Adiponectin (log 10 ng/mg), GDM: 0.199 [0.098, 0.300]; non-GDM: NR (*p* < 0.001) IGF-1 (log 10 ng/mg), GDM: 0.021 [−0.031, 0.073]; non-GDM: NR (*p* = 0.42) Leptin (log 10 ng/mg), GDM: −0.048 [−0.078, 0.174]; non-GDM: NR (*p* = 0.45)
Choi et al., [32]	The United States of America, 2021 (*n* = 189)	GDM = 35 CTL = 154	Term, no	MM (1 ± 0.2 mo, 3 ± 0.3 mo)	10:00–12:00 2 h after previous expression or breastfeed, complete right breast expression with EBP (−80 °C)	Adiponectin Insulin Leptin	ELISA	GDM: ↓ MM insulin	Adiponectin mo1 (log ng/mL), GDM: 2.90 ± 0.08; non-GDM: 2.99 ± 0.03; −0.07 (0.10) (*p* = 0.44) Adiponectin mo3 (log ng/mL), GDM: 2.65 ± 0.08; non-GDM: 2.73 ± 0.06; −0.06 (0.11) (*p* = 0.61) Insulin mo1 (log µIU/mL), GDM: 2.91 ± 0.14; non-GDM: 3.17 ± 0.06; −0.38 (0.17) (*p* = 0.03) Insulin mo3 (log µIU/mL), GDM: 2.78 ± 0.14; non-GDM: 3.18 ± 0.06; −0.53 (0.17) (*p* = 0.003) Leptin mo1 (log pg/mL), GDM: 6.44 ± 0.16; non-GDM: 6.23 ± 0.07; 0.04 (0.19) (*p* = 0.85) Leptin mo3 (log pg/mL), GDM: 6.19 ± 0.16; non-GDM: 6.03 ± 0.07; −0.01 (0.19) (*p* = 0.96)

Data are mean ± SD, median [IQR], mean difference (95% CI) and/or β (parameter estimate) (SEE). C, colostrum; CI, confidence interval; CTL, control; d, day; EBP, electrical breast pump; ECLIA, electrochemiluminescence immunoassay analyser; EIA, enzyme immunoassay; ELISA, enzyme-linked immunoassay; GDM, gestational diabetes mellitus; h, hour; HE, hand expression, HM, human milk; HPLC, high-performance liquid chromatography; IGF-1, insulin-like growth factor 1; IQR, interquartile range; MM, mature milk; mo, month; NR, not reported; RIA, radioimmunoassay; SD, standard deviation; SEE, standard error of estimate; SM, skim milk; T2D, type 2 diabetes; TM, transitional milk; ↓, lower; ↑, higher. ^a^ glycemic status assessment after pregnancy or when sample collected.

**Table 2:** Statistical analyses of relationships between maternal gestational diabetes mellitus and HM metabolic hormones.

Study	Statistical Analyses	Data Expression	Data Transformation and Adjustment for Potential Confounders, Significance Level	Total Cohort Size (Control/GDM Subgroups)	Demographics
Aydin et al., [21]	Mann–Whitney U test for comparison between groups	Mean ± SD	Correlation coefficients indicate (*p* < 0.05)	34 (14/12)	Parity, gestation, and BMI were matched
Aydin [22]	Spearman’s correlation analysis for relationship between the groups	Mean ± SD	Correlation coefficients indicate (*p* < 0.05)	20 (10/10)	Parity, gestation, and BMI were matched
Ley et al., [23]	General linear models for associations of hormones in colostrum and mature milk with prenatal maternal metabolic variables, including GDM status and time from delivery to milk collection	Mean ± SD Median [IQR] β (SEE)	Log transformed concentrations of HM components General linear model analyses with adjustment for maternal age, ethnicity, and time elapsed from birth to milk collection Correlation coefficients indicate (*p* < 0.05; *p* < 0.01 for interaction terms)	170 (133/37)	Pre-pregnancy BMI used to divide the cohort (≥25 vs. ≤25 kg/m^2^), no significant difference except in HOMA-IR and ISogtt Total of 37 women with GDM 23 ≤ 25 kg/m^2^ vs. 14 ≥ 25 kg/m^2^ (no significant difference)
Aydin et al., [24]	Mann–Whitney U test for comparison between groups	Mean ± SD	Correlation coefficients indicate (*p* < 0.05)	44 (15/15)	BMI higher in lactating women with GDM–no difference in parity and gestation
Aydin et al., [25]	Mann–Whitney U test for comparison between groups	Mean ± SD	Correlation coefficients indicate (*p* < 0.05)	36 (12/12)	BMI higher in lactating women with GDM–no difference in parity and gestation
Nunes et al., [26]	Kruskal–Wallis test with the Games–Howell post-hoc test to assess the difference between the groups	Mean ± SD Mean difference (95% CI) Median [IQR]	95% confidence intervals were considered and a significance level of 5% (*p* ≤ 0.05)	69 (21/12)	Pre-pregnancy and at birth, maternal BMI were significantly higher in GDM compared to CTL
Yu et al., [27]	Generalised Estimating Equation (GEE) using longitudinal data to assess the correlation between maternal or obstetrical factors and HM hormone concentrations	Mean ± SD Median [IQR]	Bonferroni correction to control for multiple comparisons Adjustment for maternal age Correlation coefficients indicate (*p* < 0.05)	96 (48/48)	BMI significantly higher in GDM group at pre-pregnancy and at day 90 postpartum
Fatima et al., [28]	Mann–Whitney U test for comparison between the groups	Mean ± SD	Correlation adjusted for maternal BMI	66 (33/33)	BMI significantly higher in GDM group
Ustebay et al., [29]	*t*-test and the Mann–Whitney U test for comparisons between the groups	Mean ± SD	Correlation coefficients indicate (*p* < 0.05)	53 (27/26)	Age, parity, BMI similar
Galante et al., [30]	Multivariate analysis of variance (MANOVA) used to assess the effect of categorical variables on HM composition	Mean difference (95% CI)	Log transformed concentrations of HM components Exclusive breastfeeding used as correcting factor Bonferroni correction to control for multiple comparisons Correlation coefficients indicate (*p* < 0.05)	510 (460/44)	142 women with obesity and overweight status vs. 343 women with normal weight; no details of GDM Some women did not exclusively breastfeed
Galante et al., [31]	Mixed-effects modelling used to investigate differences in HM bioactive concentrations over time across the groups defined by participant characteristics, including GDM group	Mean difference (95% CI)	Log transformed concentrations of HM components Bonferroni correction to control for multiple comparisons (*p* < 0.05)	169 (155/36)	Preterm cohort, no details of cohorts’ BMI or breastfeeding status at time of sample collection
Choi et al., [32]	Mixed-effects modelling to examine the associations of GDM status with HM hormones	Mean ± SD β (SEE)	Log transformed concentrations of HM components Adjustment for multiple covariates (*p* < 0.05)	189 (154/35)	Significantly higher BMI in GDM group

β, beta; BMI, body mass index; CTL, control; GDM, gestational diabetes mellitus; HM, human milk; HOMA-IR, homeostatic model assessment for insulin resistance; IQR, interquartile range; ISogtt, Matsuda insulin sensitivity index; SD, standard deviation; SE, standard error; SEE, standard error of estimate.

## Data Availability

All data from this study are available in the manuscript.

## Appendix A

**Table A1:** Evaluation of risk of bias in studies assessing the relationship between concentrations of metabolic hormones in human milk and gestational diabetes mellitus.

Studies	A-Selection Bias				B-Performance Bias				C-Attrition Bias				D-Detection Bias						Overall
	A1	A2	A3	O	B1	B2	B3	O	C1	C2	C3	O	D1	D2	D3	D4	D5	O	
Aydin et al., 2007 [21]	Y	Y	Y	L	Y	NA	NA	L	Y	Y	Y	L	Y	Y	Y	NA	NA	L	L
Aydin, 2010 [22]	Y	Y	Y	L	U	NA	NA	U	Y	Y	Y	L	U	Y	U	NA	NA	U	U
Ley et al., 2012 [23]	Y	N	N	H	U	NA	NA	U	Y	N	N	H	Y	Y	Y	NA	NA	L	H
Aydin et al., 2013 [24]	Y	Y	Y	L	Y	NA	NA	L	Y	Y	Y	L	Y	Y	Y	NA	NA	L	L
Aydin et al., 2013 [25]	Y	Y	Y	L	Y	NA	NA	L	Y	Y	Y	L	Y	Y	Y	NA	NA	L	L
Nunes et al., 2017 [26]	N	U	Y	H	U	NA	NA	U	Y	N	U	H	Y	Y	U	NA	NA	U	H
Yu et al., 2018 [27]	Y	Y	Y	L	Y	NA	NA	L	Y	U	U	U	Y	Y	Y	NA	NA	L	U
Fatima et al., 2019 [28]	Y	Y	Y	L	U	NA	NA	U	Y	Y	Y	L	Y	Y	Y	NA	NA	L	U
Ustebay et al., 2019 [29]	Y	Y	Y	L	Y	NA	NA	L	Y	Y	Y	L	Y	Y	U	NA	NA	U	U
Galante et al., 2020 [30]	Y	Y	U	U	U	NA	NA	U	U	Y	Y	U	Y	Y	Y	NA	NA	L	U
Galante et al., 2021 [31]	Y	N	U	H	Y	NA	NA	L	Y	U	U	U	Y	Y	Y	NA	NA	L	H
Choi et al., 2022 [32]	Y	Y	U	U	Y	NA	NA	L	Y	Y	U	L	Y	Y	Y	NA	NA	L	U

Risk of bias assessment was conducted using the National Institute for Clinical Excellence methodological checklist. H, high; L, low; N, no; NA, not applicable; O, overall; U, unclear; Y, yes.
